# A SlRBP1-*SlFBA7/SlGPIMT* module regulates fruit size in tomato

**DOI:** 10.1093/hr/uhaf089

**Published:** 2025-03-18

**Authors:** Ke Cheng, Duo Lin, Liqun Ma, Yao Lu, Jinyan Li, Guoning Zhu, Tao Lin, Guiqin Qu, Benzhong Zhu, Daqi Fu, Yunbo Luo, Hongliang Zhu

**Affiliations:** The College of Food Science and Nutritional Engineering, China Agricultural University, Beijing HaiDian District, Qinghuadonglu No.17, 100083, China; The College of Food Science and Nutritional Engineering, China Agricultural University, Beijing HaiDian District, Qinghuadonglu No.17, 100083, China; The College of Food Science and Nutritional Engineering, China Agricultural University, Beijing HaiDian District, Qinghuadonglu No.17, 100083, China; The College of Food Science and Nutritional Engineering, China Agricultural University, Beijing HaiDian District, Qinghuadonglu No.17, 100083, China; The College of Food Science and Nutritional Engineering, China Agricultural University, Beijing HaiDian District, Qinghuadonglu No.17, 100083, China; The College of Food Science and Nutritional Engineering, China Agricultural University, Beijing HaiDian District, Qinghuadonglu No.17, 100083, China; Beijing Key Laboratory of Growth and Developmental Regulation for Protected Vegetable Crops, College of Horticulture, China Agricultural University, Beijing HaiDian District, Mingyuanxilu No.2, 100193, China; The College of Food Science and Nutritional Engineering, China Agricultural University, Beijing HaiDian District, Qinghuadonglu No.17, 100083, China; The College of Food Science and Nutritional Engineering, China Agricultural University, Beijing HaiDian District, Qinghuadonglu No.17, 100083, China; The College of Food Science and Nutritional Engineering, China Agricultural University, Beijing HaiDian District, Qinghuadonglu No.17, 100083, China; The College of Food Science and Nutritional Engineering, China Agricultural University, Beijing HaiDian District, Qinghuadonglu No.17, 100083, China; The College of Food Science and Nutritional Engineering, China Agricultural University, Beijing HaiDian District, Qinghuadonglu No.17, 100083, China

## Abstract

Fleshy fruits are vital to the human diet, providing essential nutrients, such as sugars, organic acids, and dietary fibers. RNA-binding proteins play critical functions in plant development and environment adaption, but their specific contributions to fruit development remain largely unexplored. In this study, we centered on the function of SlRBP1 in tomato fruit and reported an unexpected finding that SlRBP1 controls fruit size by regulating its targets *SlFBA7* and *SlGPIMT*. Here, the fruit-specific silencing of *SlRBP1* was achieved by artificial miRNA which subsequently led to a marked reduction of fruit size. Cytological analysis suggested that *SlRBP1* silencing decreased cell division and expansion of fruit pericarp. Those key genes involved in cell development were significantly repressed in *SlRBP1* knock-down mutants. Furthermore, native RNA immunoprecipitation sequencing deciphered 83 SlRBP1-binding target RNAs in fruit, including two targets that are highly expressed in fruit: *SlFBA7* and *SlGPIMT*, which are involved in developing fruit. Indeed, silencing either *SlFBA7* or *SlGPIMT* resulted in fruit size reduction identical to that seen with *SlRBP1* silencing. These results suggest that SlRBP1 modulates fruit size through its targets *SlFBA7* and *SlGPIMT*. Our findings provide novel perspectives on the molecular mechanisms though which RNA-binding proteins control fruit size.

## Introduction

Fruit is a crucial component of the human nutritional diet, providing humans with essential amino acids, protein, dietary fiber and mineral elements and other nutrients [1]. Fruit size is closely related to fruit yield and serves as a key quality indicator for both producers and consumers. Tomato (*Solanum lycopersicum*) is one of the most grown and consumed vegetables globally. Beyond its substantial economic and nutritional value, it is also recognized as a principal model organism for investigating the developmental processes of fleshy fruits [2]. Tomato fruit development occurs in three distinct stages: fruit set, growth, and ripening [3]. Fruit size is generally influenced by several factors, including ovary size and locule number before anthesis, as well as cell division and expansion during fruit development [4].

To date, extensive research have uncovered numerous factors and genetic mechanisms that regulate tomato fruit size. Twenty-eight quantitative trait loci (QTL) contributing to fruit size have been identified in tomato by classical genetic analysis [5]. The initial QTL identified to control tomato fruit size, *fw2.2*, functioned as a suppressor of cell division, and lowering the expression of *fw2.2* causes an increase in both the size and weight of the fruit [6, 7]. *fw3.2*, also known as a P450 enzyme SlKLUH, has been shown to significantly reduce fruit and seed size when silenced [8]. Besides, *fw11.3* affects tomato fruit size by regulating cell expansion [9]. Other QTL, such as *lc*, *fas*, and *fab,* also affect fruit size by modulating the numbers of carpel [10, 11]. Beyond QTLs, hormones and transcription factors have been recognized as critical regulators influencing fruit size. For instance, during early fruit development, *SlARF9* acts as a negative regulator of cell division, and its overexpression results in significantly smaller tomatoes [12]. Whereas tomato fruits with inhibited *SlARF5* expression were significantly smaller [13]. The transcription factor *SlCDF4* is crucial in regulating gibberellin signaling pathway; its overexpression promotes both cell division and expansion, enhancing fruit yield [14]. The brassinosteroid-related transcription factor *BIM1a* has additionally been recognized as a factor that negatively regulates the expansion of pericarp cells in tomato [15]. Emerging evidence highlights the regulatory roles of numerous microRNAs in shaping fruit morphology [16]. *slmir164a* knockout mutants result in smaller fruits attributed to impaired cell division and expansion within the pericarp [17]. *SlmiR159* modulates cellular dimensions in fruits through its regulatory effects on gibberellin biosynthesis. Suppression of *SlmiR159* expression resulted in larger fruits [18]. Notwithstanding, the posttranscriptional regulation of tomato fruit size remains relatively under explored.

RNA-binding proteins (RBPs) represent a group of proteins that specifically attach to RNA, regulating RNA function either directly or indirectly. By interacting with various RNA molecules, RBPs play pivotal roles in RNA processing, editing, transport, degradation, and translation, serving as critical regulatory components in RNA biology [19]. RBPs are also essential across all stages of the plant life cycle, such as seed germination, plant growth, stress responses, and immune defenses [20, 21]. In tomato, RBPs have been implicated in leaf development and fruit quality, particularly influencing fruit shape and ripening [22]. A glycine-rich RNA-binding protein (GR-RBP), SlORRM4, is involved in fruit ripening regulation. Knockout of *SlORRM4* resulted in a substantial delay in fruit ripening coupled with reduced fruit respiration and ethylene production [23, 24]. Similarly, overexpression of the apple YTH domain-containing RNA-binding proteins *MhYTP1* and *MhYTP2* in tomato resulted in earlier ripening of tomato fruits [25]. Another RNA-binding protein, SlORRM2, significantly contributes to regulating the morphological development of tomato fruits [26]. The fruits of *Slorrm2* display pointed tips at their distal end. *SlRZ1AL, a GR-RBP* with zinc-finger motif, has also been reported to participate in regulating carotenoid biosynthesis, impacting tomato fruit ripening. Knockout of *SlRZ1AL* resulted in reduced fruit lycopene content and fruit weight, with downregulation of genes encoding enzymes crucial for carotenoid biosynthesis and metabolism [27]. However, the role of *SlRZ1AL* in regulating fruit size has not been elucidated. In fact, no mechanism by which RBP regulates fruit size has been reported so far.

In previous study, we identified a GR-RBP, SlRBP1, which interacts with the eukaryotic translation initiation factor SleIF4A2 to regulate the translation efficiency of key photosynthesis-associated mRNAs, thereby affecting the structure and function of chloroplasts [28]. Knockdown of *SlRBP1* by artificial miRNA resulted in tomato plants exhibiting dwarfism, yellowing leaves, reduced photosynthetic capacity, and decreased fruit size [28]. However, whether the smaller fruit size was due to impaired photosynthesis and vegetative development or a direct regulatory role of SlRBP1 in fruit development which still remains ambiguous.

Here, we specifically silenced *SlRBP1* in tomato fruit using the phosphoenolpyruvate carboxylase *PPC2* promoter and observed a consistent reduction in fruit size, independent of plant vegetative development. Cytological analysis further revealed that SlRBP1 influences pericarp development by regulating cell division and expansion. Further, RIP-seq revealed that two target genes involved in fruit size development, *SlFBA7* and *SlGPIMT*, were stably bound to SlRBP1. Similar to the situation in leaves, SlRBP1 interacts with SleIF4A2 in the fruit, thereby regulating the level of targets translation. Additionally, the silencing of either *SlFBA7* or *SlGPIMT* led to a remarkably smaller fruit size. Based on these results, these findings offer novel perspectives on the posttranscriptional regulatory mechanisms mediated by SlRBP1 that govern tomato fruit size.

## Result

### Fruit-specific silencing of *SlRBP1* reduces tomato fruit size

In this study, we performed a detailed examination of *SlRBP1* expression patterns of different tissue and development stage in tomato (Fig. S1a), revealing consistently higher expression levels in all stages of fruit compared to leaves, with peaks at 20 days postanthesis (DPA). These results imply that *SlRBP1* may play a critical role in tomato fruit development. Previous studies have reported that silencing of *SlRBP1* using the constitutive 35S promoter resulted in dwarf plants, yellowed leaves, flower abscission, and smaller, unevenly colored fruits [28]. These pleiotropic phenotypes hindered precise evaluation of specific role of *SlRBP1* in fruit development. To minimize developmental disruptions and accurately assess fruit phenotypes, we utilized the fruit-specific PPC2 promoter [29] to either overexpress (PPC2pro::*SlRBP1*, abbreviated as OE-*SlRBP1*) or silence (PPC2pro::amiR-*SlRBP1*, abbreviated as amiR-*SlRBP1*) *SlRBP1* in the tomato fruit.

**Figure 1:**
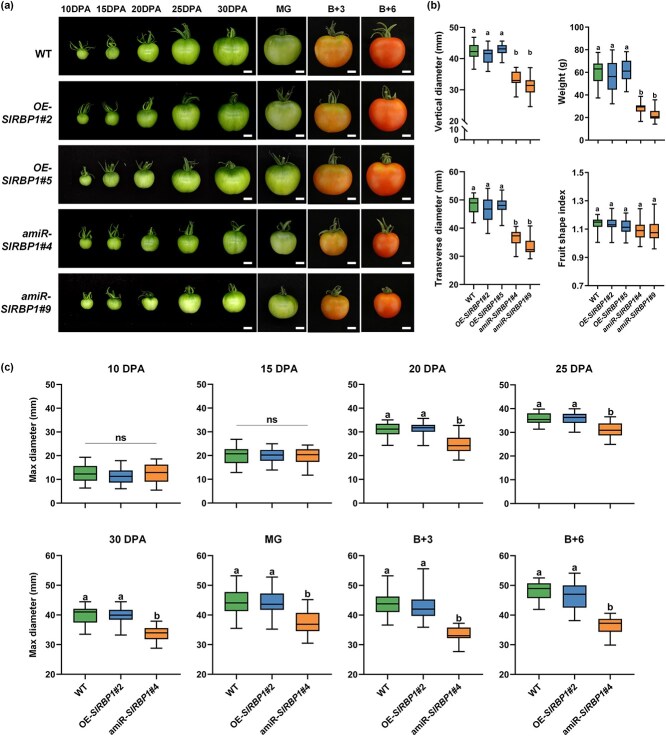
Specific silencing of *SlRBP1* in fruit results in smaller tomato fruit. (a) Phenotypic comparison of WT, OE-*SlRBP*1#2, OE-*SlRBP1*#5, amiR-*SlRBP1*#4 and amiR-*SlRBP1*#9 fruits in different stages. (b) Weight, longitudinal/transverse diameters, and shape index statistics of WT, OE-*SlRBP1*#2, OE-*SlRBP1*#5, amiR-*SlRBP1*#4 and amiR-*SlRBP1*#9T1 lines B + 6 fruits. *n* = 30. (c) Maximum width of the WT, OE-*SlRBP1* and amiR-*SlRBP1* fruits at different development stages. *n* = 30. Different letters represent significant differences (*P* < 0.05). DPA, days post anthesis; MG, mature green stage; B + 3, 3 days after breaker; B + 6, 6 days after breaker. Scale bars = 1 cm.

Since the *SlPPC2* gene exhibits peak expression during the mature green (MG) stage (Fig. S1b), MG fruits were selected for comparing *SlRBP1* transcript levels between wild type (WT) and *SlRBP1* transgenic plants. There were nine lines with a significant increase in SlRBP1 expression as OE-*SlRBP1*, while 13 amiR-*SlRBP1* lines displayed varying degrees of reduced expression (Fig. S1c). In addition, the Myc tagged SlRBP1 fusion protein was detected in the corresponding OE-*SlRBP1* lines, but not in wild type (Fig. S1d). Based on the most accumulation in *SlRBP1* transcript levels relative to WT, we selected OE-*SlRBP1*#2, OE-*SlRBP1*#5, amiR-*SlRBP1#4*, and amiR-*SlRBP1*#9 for further observations. The fruits of amiR-*SlRBP*1#4 and amiR-*SlRBP1*#9 were much smaller than those of the WT from 20 DPA until B + 6 (6 days postbreaker), while the fruits of OE-*SlRBP1*#2 and OE-*SlRBP1*#5 were slightly larger but not significantly different from WT (Fig. 1a). To further characterize these differences, morphological traits of mature red fruits (including weight, longitudinal/transverse diameters, and shape index) were quantitatively assessed. Statistical analysis revealed that amiR-*SlRBP1* lines exhibited significantly reduced fruit dimensions (length, width) and weight compared to WT controls (Fig. 1b). Meanwhile, the fruit shape index remained relatively consistent across all lines (Fig. 1b), indicating that the observed variations primarily affected size rather than shape. To identify the developmental stage at which *SlRBP1* begins to exert its influence, we measured fruit size at various time points, including 10, 15, 20, 25, and 30 DPA, as well as at the MG, B + 3 (3 days postbreaker), B + 6 stages of WT, OE-*SlRBP1* and amiR-*SlRBP1* plants. The results showed little significant differences in fruit size among WT, OE-*SlRBP1* and amiR-*SlRBP1* at 10 and 15 DPA. After 15 DPA, the fruit entered a phase of rapid growth. From 20 DPA onwards, amiR-*SlRBP1* exhibited slower development, resulting in significantly smaller fruits compared to WT and OE-*SlRBP1*. By the end of the B + 6 stage, the final fruit transverse diameter of amiR-*SlRBP1* was 10 to 25 mm smaller than that of WT and OE-*SlRBP1* (Fig. 1c).

We also assessed the vegetative development of WT, OE-*SlRBP1*, and amiR-*SlRBP1* plants (Fig. S2). The plant height, leaf, flowers, and inflorescences development of all lines were consistent, with little significant differences (Fig. S2a–e). RT-qPCR analysis confirmed that *SlRBP1* expression alterations were restricted to the fruit (Figs S1a and S2f–h). What is more, we evaluated the impact of SlRBP1 on fruit ripening and quality. The results indicated little differences in the onset of color break, the ripening process, soluble solids, and titratable acid content among WT, OE-*SlRBP1*, and amiR-*SlRBP1* (Fig. S3). In summary, these results indicate that overexpressing or silencing of *SlRBP1* in fruit-specific pattern does not impact plant vegetative growth or fruit quality, confirming that specific silencing of *SlRBP1* leads to a significant reduction in tomato fruit size without affecting fruit shape and quality.

### SlRBP1 regulates fruit size by controlling cell division and expansion

**Figure 2:**
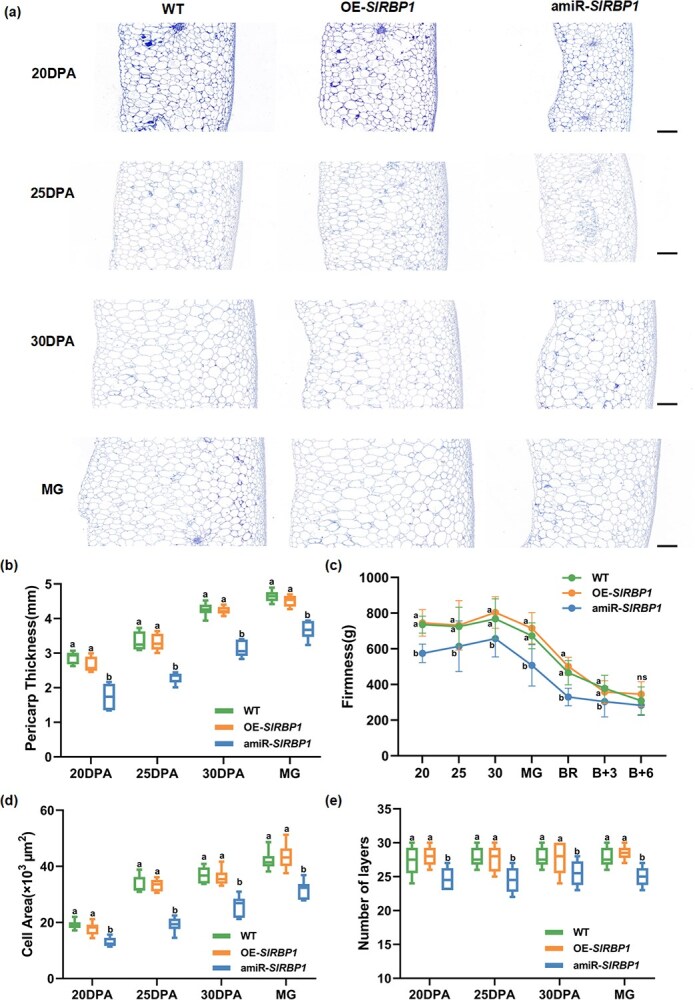
Silencing *SlRBP1* affects tomato pericarp development. (a) Representative images of pericarp cells of WT, OE-*SlRBP1* and amiR-*SlRBP1* fruits at different stages. Scale bars =500 μm. MG, mature green stage. DPA, days post anthesis. (b-e), Measurement of pericarp thickness (b), fruit pericarp firmness (c), pericarp cell size (d) and pericarp cell layer number (e) of WT, OE-*SlRBP1* and amiR-*SlRBP1* fruits at different development stages. Different letters represent significant differences (*P* < 0.05). B + 3, three days after breaker; B + 6, six days after breaker.

To further elucidate how *SlRBP1* influences fruit size, we examined peel thickness at periods of variation in fruit size (20, 25, 30 DPA, and MG) among WT, OE-*SlRBP1*, and amiR-*SlRBP1* lines (Fig. 2a). Consistent with the observed fruit size changes, pericarp thickness in amiR-*SlRBP1* was thinner than that of WT and OE-*SlRBP1* across all stages (Fig. 2b). Concertedly, we assessed fruit firmness from 20 DPA to B + 6. At 20, 25, 30 DPA, MG, BR, and B + 3, the fruit firmness of amiR-*SlRBP1* was markedly lower in comparison to that of WT and OE-*SlRBP1*. However, at the B + 6 stage, no significant differences were detected among the three lines. This might be attributable to the circumstance that all the fruits had reached an overmature state by this time (Fig. 2c).

**Figure 3:**
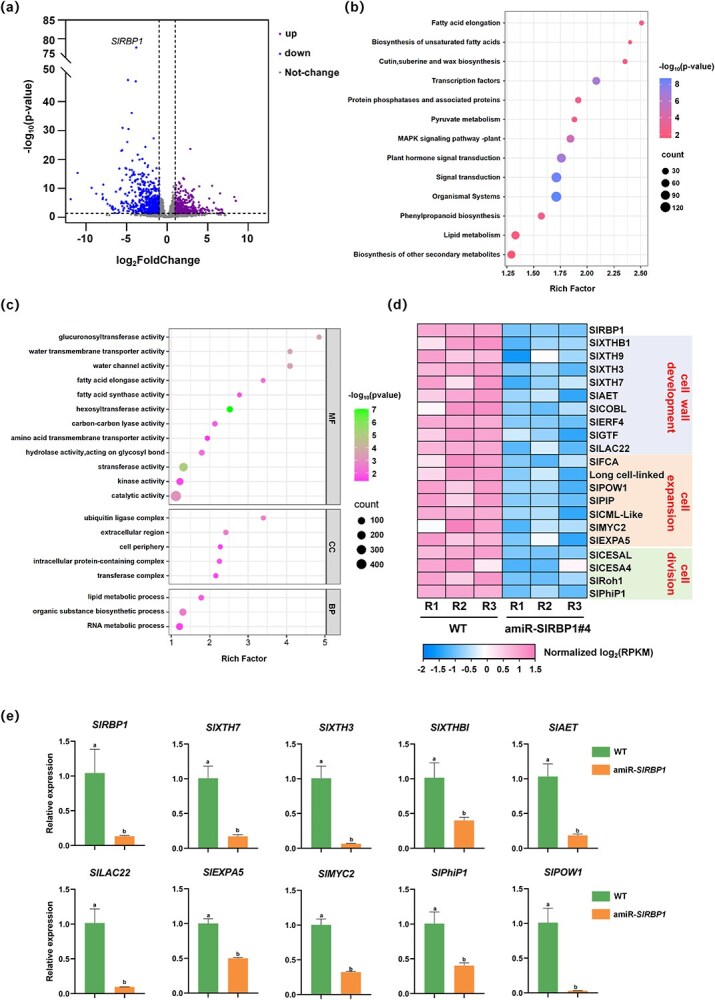
SlRBP1 affects the expression of key genes related to cell development. (a) Volcano plot of RNA-seq data between WT and amiR-*SlRBP1* 25 DPA fruits. Each dot represents a DEG. up, upregulated genes; down, downregulated genes. Thresholds (|FC| > 2, adjusted *P* < 0.05) are marked by dotted lines. GO enrichment (b) and KEGG enrichment analysis (c) of DEGs in amiR-*SlRBP1* 25 DPA fruits compared to WT. (d) Heat map visualization of downregulated cell development-related gene transcripts in amiR-*SlRBP1* versus WT fruits. (e) RT-qPCR verifies the expression of cell development related genes in WT and amiR-*SlRBP1* 25DPA fruits. *Actin* was reference gene. Data represents mean ± SD of three biological replicates. Different letters represent significant differences (*P* < 0.05).

In addition, we analyzed the cell area and the quantity of cell layers at 20, 25, and 30 DPA, along with the MG stage (Fig. 2d and e). In amiR-*SlRBP1*, the average cell area was significantly smaller compared to WT, and the number of cell layers was reduced by approximately four layers, whereas both cell area and cell layer number in OE-*SlRBP1* were comparable to WT, showing little significant differences. In addition, in order to preclude the potential impacts of DNA ploidy and hormones on cell size and quantity, we assessed of the DNA ploidy (Fig. S4) and hormone (Fig. S5) levels within the pericarp cells of three distinct lines. The experimental outcomes demonstrated that there were no substantial disparities in DNA ploidy and hormone levels among the WT, OE-*SlRBP1*, and amiR-*SlRBP1* fruits at 25 DPA. Taken together, the cytomorphological results imply that *SlRBP1* exerts an impact on both the pericarp cell layer numbers and cell areas by regulating both cell division and expansion processes. Consequently, it contributes to the control of fruit size, independent of ploidy, as well as hormone levels.

### Silencing of *SlRBP1* in fruit impaired the expression of key cell development-related genes

In order to further explore the molecular mechanism by which *SlRBP1* regulates fruit size, we performed a comparison of analysis between WT and amiR-*SlRBP1* fruits at 25 DPA, a stage where the difference in fruit size is most pronounced. The RNA-seq results revealed 723 genes significantly downregulated and 542 genes significantly upregulated in amiR-*SlRBP1* fruits compared to WT (|FoldChange| > 2, adjusted *P* < 0.05; Fig. 3a; Table S1). KEGG pathway analysis further highlighted significant alterations in biological pathways, including fatty acid elongation, cutin, suberine, and wax biosynthesis, plant hormone signal transduction, and lipid metabolism (Fig. 3b). GO enrichment analysis (Fig. 3c) indicated that these differentially expressed genes (DEGs) were primarily associated with fatty acid synthase and elongase activities, water channel activity (molecular functions), ubiquitin ligase complexes (cellular components), and lipid metabolic processes (biological processes). Related genes in these pathways have been reported to be involved in fruit size [30, 31].

Additionally, we screened highly distinct DEGs with |FoldChange| > 8 and adjust *P* < 0.05. The analysis showed that several genes associated with cell wall development, cell expansion, and cell division were significantly downregulated in amiR-*SlRBP1* (Fig. 3d). Specifically, cell wall development-related genes, including *SlXTH3*, *SlXTH7*, *SlXTHB1* (xyloglucan endo-transglycosylase family), *SlAET* (AE family transporter protein), and *SlLAC22* (Laccase-22), as well as cell expansion-related genes like *SlMYC2* (basic helix–loop–helix transcription factor) and *SlEXPA5* (Expansin), and the cell division-related gene *SlPhIP1* (Phi-1 protein), were notably downregulated. The expression levels of these genes in WT and amiR-*SlRBP1* fruits at 25 DPA were further validated using RT-qPCR (Fig. 3e). These findings intimate that *SlRBP1* plays a critical role in orchestrating the transcriptional activity of core cell development-related genes.

### Direct targets of SlRBP1 in fruit were closely related to tomato fruit development

**Figure 4:**
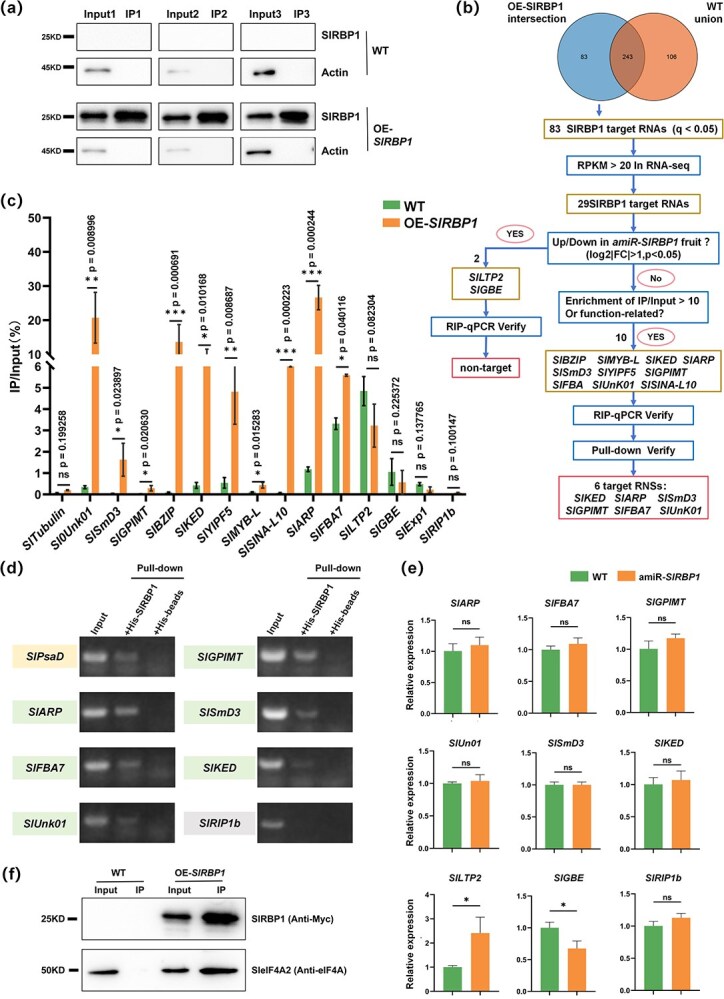
SlRBP1 specifically binds to target RNAs which are related to fruit development. (a) Western Blot of SlRBP1 protein accumulation in Input and IP samples from WT and OE-*SlRBP1* 25 DPA fruits. C-Myc antibody was used to detect the fusion proteins. *Actin* as internal reference protein. (b) A comprehensive schematic diagram illustrating the workflow for identifying potential SlRBP1-target transcripts in 25 DPA fruits. (c) Native RNA immunoprecipitation(nRIP) combined with RT-qPCR verified the binding of SlRBP1 to the targets in 25 DPA fruit. The percentage represents the ratio of IP-enriched RNA compared to input sample. Error bars represent ±SD over biological triplicates. *SlTublin*, *SlRIP1b*, and *Slexp1* were utilized as negative controls. (d) Protein pulldown verified the binding of SlRBP1 and target genes *in vitro*. His-SlRBP1 was purified and incubated with total RNA of 25 DPA fruit, and candidate targets were detected by PCR. *SlRIP1b* was used as negative control. *SlPsaD* served as positive control that has been validated in previous study. (e) Expression of SlRBP1-binding targets RNA in WT and amiR-*SlRBP1*. *SlGBE* and *SlLTP2*, target genes that vary in RNA-seq of amiR-SlRBP1. *SlARP*, *SlFBA7*, *SlGPIMT*, *SlUn01*, *SlSmD3*, and *SlKED* are target genes that not changed in RNA-seq of amiR-*SlRBP1*. *Actin* was reference gene. Error bars indicate ±SD over three biological replicates. (f) SlRBP1 interacted with SleIF4A2 *in vivo*. Immunoprecipitation was carried out using OE-*SlRBP1* and WT 25 DPA fruit. Asterisks indicate significant difference (^*^P < 0.05, ^**^P < 0.01, ^***^P < 0.001; ns, not significant).

RNA-binding proteins rely on specific target RNAs to execute their functional activities. To identify the *in vivo* targets of SlRBP1, we performed native RNA immunoprecipitation (nRIP) using 25 DPA fruits from the OE-*SlRBP1* line, with WT fruits serving as negative controls. Immunoblot analysis revealed substantial accumulation of Myc-SlRBP1 in immunoprecipitated (IP) sample versus input, validating successful isolation of the target protein (Fig. 4a). Under stringent screening conditions (*q* < 0.05) for nRIP-seq analysis, we identified 83 transcripts that were significantly enriched in OE-*SlRBP1* but absent in WT (Fig. 4b). Of these, we selected 29 genes based on their high expression levels (RPKM >20) for further analysis (Table S2). Comparison with DEGs in amiR-*SlRBP1* revealed that only two targets, *SlLTP2* (nonspecific lipid-transfer protein 2) and *SlGBE* (1,4-alpha-glucan branching enzyme), exhibited altered transcription levels. However, nRIP-qPCR analysis did not show significant enrichment of these two genes *in vitro* compared to WT (Fig. 4c).

Next, based on gene enrichment and functional relevance, we identified 10 target genes with higher enrichment (fold enrichment >10) or known functional roles. We then assessed the binding affinity of these potential targets to SlRBP1 both *in vivo* and *in vitro*. nRIP-qPCR results revealed marked enrichment of the selected targets in the OE-SlRBP1 IP compared to the WT, while nontarget genes, such as *SlTubulin*, *SlExp1*, and *SlRIP1b* [23], served as negative controls (Fig. 4c). Furthermore, protein pull-down assays confirmed that several genes, including *SlARP* (Auxin-repressed protein), *SlFBA7* (fructose-bisphosphate aldolase 7), *SlUn01* (unknown protein), *SlGPIMT* (GPI mannosyltransferase 1), *SlSmD3* (small nuclear ribonucleoprotein SmD3), and *SlKED*, directly bind to SlRBP1 *in vitro* (Fig. 4d). In conclusion, we identified six targets - *SlARP*, *SlFBA7*, *SlUn01*, *SlGPIMT*, *SlSmD3*, and *SlKED* - that bind to SlRBP1 both *in vivo* and *in vitro*, confirming them as bona fide targets of SlRBP1 rather than products of nonspecific interactions with the Myc beads fusion. Expression pattern analysis indicated that the transcriptional activities of *SlARP*, *SlFBA7*, *SlGPIMT*, and *SlUn01* were substantially elevated during fruit development compared to those in nonreproductive tissues, which implies their potentially crucial role in fruit development (Fig. S6).

Given that previous study has been illustrated that SlRBP1 and SleIF4A2 interact within the leaf, thereby modulating the translational activity of targets [28]. Herein, we postulate that this model is also applicable in the fruit. Therefore, we assessed the transcript levels of these targets in WT and ami-*SlRBP1* 25DPA fruits*.* RT-qPCR analysis confirmed the expression level of these genes in WT and amiR-*SlRBP1* lines were similar (Fig. 4e). Concurrently, no alternative splicing occurred in these target genes (Table S3), which further supported their regulatory relationship with SlRBP1. Furthermore, we conducted immunoprecipitation *in vivo* with 25 DPA OE-*SlRBP1* fruits and evidenced that SlRBP1 also specifically interacts with SleIF4A2 within the fruit (Fig. 4f). Overall, our findings proffer that SlRBP1 modulates fruit size through the modulation of target genes related to fruit development at the translational level rather than at the transcriptional level.

### Silencing of the SlRBP1 targets *SlFBA7* and *SlGPIMT,* respectively, decreases fruit size of tomato

**Figure 5:**
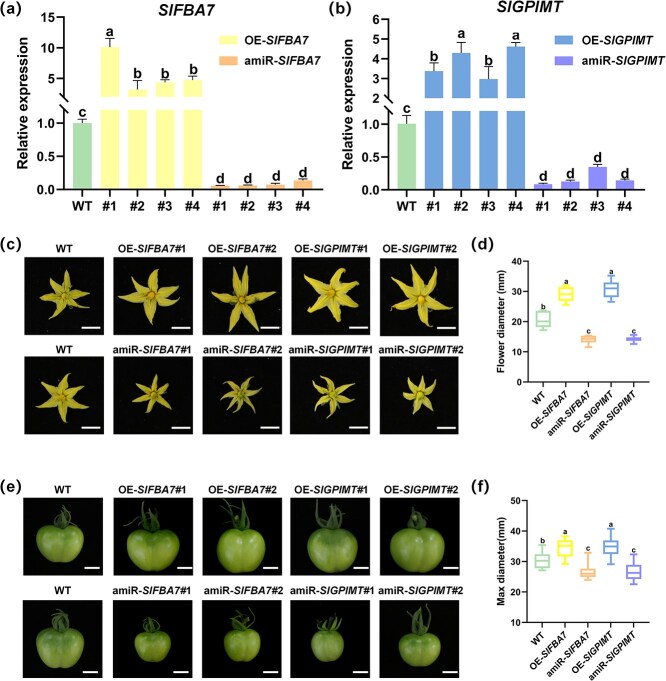
*SlFBA7* and *SlGPIMT* positively regulate fruit size in tomato. (a) Quantitation of *SlFBA7* in T0 generation over-expressed or artificial miRNA silenced transgenic plants leaf. Relative expression of *SlFBA7* in WT was set to 1. (b) Quantitation of *SlGPIMT* in T0 generation over-expressed or artificial miRNA silenced transgenic plants leaf. Relative expression of *SlGPIMT* in WT was set to 1. *Actin* was reference gene. Error bars indicate ±SD over three biological replicates. (c) Phenotypic comparison of WT, OE-*SlFBA7*, amiR-*SlFBA7*, OE-*SlGPIMT*, and amiR-*SlGPIMT* in flower. Scale bars = 1 cm. (d) The diameter of flower in WT, OE-*SlFBA7*, amiR-*SlFBA7*, OE-*SlGPIMT,* and amiR-*SlGPIMT* plants. *n* = 15. (e) Phenotypic comparison of WT, OE-*SlFBA7*, amiR-*SlFBA7*, OE-*SlGPIMT*, and amiR-*SlGPIMT* in 25DPA fruits. Scale bars = 1 cm. (f) Maximum width of WT, OE-*SlFBA7*, amiR-S*lFBA7*, OE-*SlGPIMT*, and amiR-*SlGPIMT* 25 DPA fruits. *n* = 20. Different letters represent significant differences (*P* < 0.05).

To further investigate the role of SlRBP1 target genes in regulating fruit size, we selected *SlFBA7* and *SlGPIMT* for transgenic manipulation. Driven by the CaMV 35S promoter, we generated over-expressed (OE) and artificial miRNA silenced (amiR) transgenic plants, resulting in the genotypes OE-*SlFBA7*, amiR-*SlFBA7*, OE-*SlGPIMT*, and amiR-*SlGPIMT*, respectively. Initial RT-qPCR analysis confirmed successful transformation, with four distinct independent transgenic lines generated for each genotype (Fig. 5a and b). Two lines per genotype were subsequently chosen for further analysis.

Encouragingly, OE-*SlFBA7* or OE-*SlGPIMT* plants presented larger flowers and fruits than WT. Conversely, silencing *SlFBA7* or *SlGPIMT* resulted in smaller flowers (Fig. 5c) as data shown in Fig. 5d. Besides, we also determined the size of OE-*SlFBA7*, amiR-*SlFBA7*, OE-*SlGPIMT*, and amiR-*SlGPIMT* tomato fruits at 25 DPA. Unsurprisingly, tomato fruits overexpressing *SlFBA7* and *SlGPIMT* were larger than WT, whereas those fruits with silencing of *SlFBA7* and *SlGPIMT* were significantly smaller, which was consistent with the amiR-*SlRBP1* phenotype (Fig. 5e and f). Altogether, these results indicate that *SlFBA7* and *SlGPIMT* act as positive regulators in relation to tomato fruit size. Moreover, SlRBP1 directly associates with these targets so as to maintain an appropriate fruit size in tomato.

## Discussion

Fruit size is a critical quality attribute, traditionally regulated by quantitative trait loci and transcription factors [4]. However, posttranscriptional regulators of fruit size remain largely unexplored. In this study, we identified a GR-RBP, SlRBP1, that modulates tomato fruit size at the translation level. Specific silencing *SlRBP1* in fruit resulted in a marked reduction in fruit size starting at 20 DPA, which persisted throughout development and ripening (Fig. 1). This reduction was accompanied by a significant decrease in the number of pericarp cell layers and cell area, indicating that *SlRBP1* regulates both cell division and expansion (Fig. 2). On the contrary, although cell division is known to dominate early fruit development (0–8 DPA) [32], we observed little size changes in silenced fruits at 10 DPA. This could be attributed to the difficulty of accurately measuring small fruits at early stages or the relatively low *SlRBP1* expression at 10 DPA. Undoubtedly, our results demonstrate that silencing *SlRBP1* significantly reduces fruit size, which could directly impact yield. Interestingly, essential fruit quality traits, such as soluble solids, titratable acidity, and ripening progression, exhibited little significant alterations in amiR-*SlRBP1* fruits (Fig. S3). This indicates that SlRBP1 specifically regulates fruit size without compromising quality traits, highlighting its potential as a promising target for yield improvement in breeding programs. Interestingly, overexpression of *SlRBP1* did not result in larger fruits, despite successful transgene integration and elevated *SlRBP1* expression levels in fruit tissues (Fig. S6). This observation suggests the presence of a feedback regulatory mechanism that limits the effects of *SlRBP1* overexpression [33], thereby preventing excessive fruit growth even under conditions of *SlRBP1* abundance. These findings collectively imply that *SlRBP1* is part of a finely tuned regulatory network that ensures balanced fruit development. In addition to its role in fruit size regulation, silencing *SlRBP1* led to thinner fruit skin and reduced fruit firmness, traits that may increase susceptibility to mechanical damage and spoilage. This suggests that manipulating *SlRBP1* could influence not only yield but also fruit texture and shelf life. Furthermore, *SlRBP1* may play a role in the biosynthesis of secondary metabolites, such as carotenoids and flavonoids, which are critical for fruit color, flavor, and nutritional value. For instance, transcriptome analysis revealed the downregulation of lipid metabolism-related genes (Fig. 3c), which could indirectly affect carotenoid production [34, 35]. In future studies, a detailed investigation into the changes in secondary metabolites in *amiR-SlRBP1* fruits will provide critical insights into the broader effects of *SlRBP1* manipulation on fruit quality.

What is more, transcriptome data analysis of WT and amiR-*SlRBP1* showed that a large number of genes related to cell wall development, cell expansion and cell division were significantly downregulated (Fig. 3). For example, the recently reported transcription factor SlMYC2 and its target *SlEXPA5* positively regulate the fruit size by promoting cell expansion in tomato fruits [36]. Moreover, we identified six targets bound by SlRBP1 *in vivo* and *in vitro*. And these target genes related to fruit development were modulated independent of transcriptional level (Fig. 4). Similarly, in our previous work, we identified 218 SlRBP1-bound target genes in leaves, yet only five showed altered transcription levels in the *SlRBP1* knockdown line. And also, these five genes did not successfully bind to SlRBP1 *in vitro*, further confirming that the transcriptional levels of true SlRBP1 targets remain unchanged (Ma *et al.*, 2022). Likewise, we confirmed that SlRBP1 interacts with SleIF4A2 in fruit in this study. This pattern observed in both leaves and fruit suggests that SlRBP1 primarily exerts its regulatory function at the translational level, influencing the expression of its target genes in a tissue-specific manner, thereby contributing to distinct physiological phenotypes.

Surprisingly, when we obtained over-expressed or silenced transgenic plants of the downstream targets *SlFBA7* and *SlGPIMT*, all these mutants displayed dramatic phenotypes on fruit sizes. Tomato fruits silenced by *SlFBA7* or S*lGPIMT* were significantly smaller than WT, which was consistent with the phenotype of silenced SlRBP1 (Fig. 5). Based on the aforementioned findings, we developed a model concerning the regulation of fruit size by SlRBP1. SlRBP1 binds with the positive regulators of fruit size, namely *SlFBA7* and *SlGPIMT*, and modulates their translational levels for the purpose of regulating fruit size.

Fructose-1,6-bisphosphate aldolase (FBA) is a crucial enzyme in energy metabolism, facilitates the reversible transformation of fructose-1,6-bisphosphate (FBP) into dihydroxyacetone phosphate (DHAP) and glyceraldehyde 3-phosphate (G3P) within the glycolytic pathway [37]. FBA plays a critical role in numerous essential physiological and biochemical pathways, including the fixation of CO_2_, regulation of secondary metabolism and plant development [38]. Studies have shown that FBA plays an important role in bamboo tissue elongation. *SlFBA7* overexpression increased seed size and stem diameter [39]. These results suggest that FBA may play a role in cell expansion. Meanwhile, *SlGPIMT*, a glycosylphosphatidylinositol mannosyltransferase, is highly expressed in the early stages of fruit development and is primarily responsible for transferring the first alpha-1,4-mannose unit, facilitating GPI precursor assembly [40]. Knockout of its homolog in *Arabidopsis* resulted in decreased crystalline cellulose in the cell wall and irregular deposition of xyloglucan and callose, underscoring its critical function in cell wall integrity [41]. Our findings demonstrate that SlRBP1 regulates the translational efficiency of *SlFBA7*/*SlGPIMT*, key downstream target genes governing fruit size. Reduced protein-level expression of these targets could alter downstream transcriptional cascades, potentially establishing a feedback regulatory loop that suppresses the expression of cell development-related genes. Therefore, the downregulation of genes associated with cell wall development, cell expansion, and cell division may represent an indirect consequence of *SlRBP1* silencing. However, the molecular mechanisms underlying how *SlFBA7* and *SlGPIMT* regulate fruit size need to be further investigation in the subsequent studies.

And also, our analysis identified several transcription factors, such as members of the MYB-like and BZIP families, as putative SlRBP1 targets in fruit (Fig. 4c). This suggests that SlRBP1 may play a role in modulating transcriptional networks through its interactions with these transcriptional regulators. RNA-binding proteins act as part of a broader posttranscriptional regulatory network. SlRBP1 may collaborate with other RNA-binding proteins that regulate RNA localization, stability, or translation, to fine-tune the expression of genes critical for fruit development. This implies that SlRBP1 may not function in isolation but rather in concert with other regulatory factors to orchestrate the posttranscriptional regulation of fruit growth. In addition to its role in RNA regulation, emerging evidence suggests that RBPs can also bind to chromatin, directly influencing transcriptional regulation [42]. If SlRBP1 interacts with chromatin regions, it may have a dual role in both posttranscriptional and transcriptional regulation of genes involved in fruit development.

Comparison with previous studies, the interaction between SlRBP1 and the translation initiation factor SleIF4A2 observed in our study aligns with previous research conducted in leaves, where SlRBP1 was shown to regulate the translation of photosynthesis-related mRNAs. This consistent finding suggests that SlRBP1 employs a conserved translational regulatory mechanism across different tissues, including fruit. This highlights the broader functional role of SlRBP1 in regulating gene expression at the translational level. Besides, while most studies on fruit size have predominantly focused on QTLs and transcription factors, our work underscores the significant role of RBPs, like SlRBP1, in the translation regulation of fruit size. However, this study does not fully capture the relationship between SlRBP1 targets mRNA and protein levels. Proteomics analysis would provide a more comprehensive understanding of how SlRBP1 regulates its targets at the translational level in future studies.

Notably, to describe fruit phenotypes without extra developmental disruptions, this study employed the fruit-specific *PPC2* promoter to over-express or silence *SlRBP1*. our findings revealed distinct targets in fruit tissues compared to those identified in leaves, uncovering a previously unrecognized role of *SlRBP1* in regulating fruit size. This discovery offers a promising strategy for the precise dissection of gene function, particularly in the context of pleiotropic and lethal genes. In biological systems, a single gene frequently influences multiple phenotypic traits—a phenomenon known as pleiotropy [43, 44]. Consequently, the regulation of gene expression via gene-editing techniques or constitutive promoters can lead to undesirable outcomes, including plant lethality or disruptions in various developmental and physiological processes. In contrast, tissue-specific promoters enable targeted investigations of gene functions within defined tissues or developmental stages, thereby minimizing pleiotropic complications [45]. Moreover, this approach has significant potential for advancing crop breeding initiatives. For instance, while the knockdown of *SlWAT1* in tomato enhances resistance to *Vascular Wilt Fungi*, it simultaneously results in severe growth and developmental impairments [46]. By using suitable tissue-specific promoters, it is expected to produce tomato varieties that are resistant to the disease and do not affect growth and development. Nowadays, a number of tomato fruit-specific promoters have already been explored [29], and future research could focus on expanding the array of tissue-specific promoters. These tools hold promise for advancing genetic manipulation techniques and supporting the improvement of crop traits in breeding.

In conclusion, this study significantly advances our understanding of the genetic networks underlying fruit development by uncovering a novel posttranscriptional regulatory mechanism mediated by *SlRBP1* in controlling tomato fruit size. These findings not only elucidate a key molecular pathway governing fruit size determination but also emphasize the broader functional importance of RBPs in plant biology, particularly in the context of fruit development. Importantly, our work provides the first mechanistic evidence that *SlRBP1* regulates fruit size through translational control, thereby expanding the known functional repertoire of plant RBPs. By establishing a direct link between RNA metabolism and organ size regulation, this study bridges a critical knowledge gap in fruit developmental biology and opens new avenues for exploring the role of posttranscriptional regulation in plant growth and development. Besides, we introduce a novel and effective strategy for the precise functional characterization of genes, particularly those with pleiotropic effects. The implications of this research extend to breeding strategies for high-yield, high-quality tomato varieties, and offer a foundation for improving traits in other crops.

## Materials and methods

### Plant materials and growth conditions

The tomato variety used in this study is Ailsa Craig. All plants were cultivated in growth chambers at 25°C day/20°C night temperature under 16-hour light/8-hour dark cycle. At anthesis, the fruits were labeled 0 DPA. The fruits were harvested at 25DPA, mature green (MG), breaker (Br), 3 days postbreaker (B + 3), 6 days postbreaker (B + 6), 9 days postbreaker (B + 9). Samples are frozen in liquid nitrogen immediately after harvest and then stored at −80°C.

### Plasmid construction and tomato transformations

The pCAMBIA1300-Flag-Myc-SlRBP1 and pCAMBIA1300-amiR-SlRBP1 were constructed in the previous article [28]. The *SlRBP1* native promoter in the pCAMBIA1300-Flag-Myc-*SlRBP1* was replaced with the phosphoenolpyruvate carboxylase (*PPC2*) promoter. To construct the amiR-*SlRBP1* vector, The CaMV 35S promoter in pCAMBIA1300-amiR-*SlRBP1* was replaced with the *PPC2* promoter. The final binary vectors were introduced to GV3101 and then transformed into tomato cotyledons using previously described methods [47].

The amplification of all sequences was performed utilizing Phanta Max Super-Fidelity DNA Polymerase (Vazyme, Nanjing, China), and subsequently cloned with the ClonExpress II One Step Cloning Kit (Vazyme, China). All primers for plasmid construction are listed in Table S4.

### Phenotypic analysis of fruits

The fruits used to measure fruit weight, maximum width, fruit shape index, transverse and longitudinal diameter of all transgenic fruit and WT were sampled from fifteen plants as the biological replicates. Each line has more than 30 fruit quality indicators measured. The height of a plant is the distance from the lowest point to the highest point of growth after four ears of flowers.

### Measurement of soluble solids

To examine sugar and titratable acid, fruits were harvested at MG, Br, B + 3, B + 6, and B + 9 stages. Juice extracted from fruits at different stage was measured using a PAL-1 digital sugar meter (ATAGO, Tokyo, Japan) to obtain the soluble solids content. Nine fruits were measured for each sample as biological replicates.

### Measurement of titrable acid

The titration method was employed to determine the titratable acidity (TTA) content of tomato fruits as described previously [48]. Nine fruits were measured for each sample as biological replicates.

### Measurement of firmness

The firmness of each fruit was measured on three sides of the pericarp was measured by TA.XT Plus texture analyzer (Surrey, United Kingdom). Nine fruits were measured for each sample as biological replicates.

### Histological analyses

Approximately 100 cubic millimeters of pericarp were excised from the equatorial region of transversely crossed fruits immediately after fixation with FAA solution (Coolaber, Beijing, China), followed by paraffin embedding. Staining of pericarp sections using toluidine blue. WT and amiR-*SlRBP1* each took four fruits for biological repetition. The scanning of the paraffin sections was done by Servicebio (Wuhan, China). The pericarp thickness, numbers of cell layer and cell areas were calculated using the ImageJ (version 1.54d).

### Flow cytometry analysis

The 25 days of fruits were collected for flow cytometry measurements. CyStain UV Precise P extraction buffer (Sysmex Partec, Goerlitz, Germany, code 05-5002) was used to extract nuclei. Each analysis encompassed 10 000 nuclei. Flow cytometry data was Flow cytometry data were determined by Golden Intelligence Biotechnology Co. (Beijing, China).

### Measurement of hormone

The hormone of 25 DPA fruit was determined by liquid chromatography (KuoGangJian Biotechnology, Taian, China). Nine fruits were measured for each sample as biological replicates.

### RNA extraction and RT-qPCR

Leaf and fruit RNAs were extracted as in previous studies [47]. The cDNA was synthesized using the HiScript II 1st Strand cDNA Synthesis Kit (Vazyme, China). RT-qPCR was carried out using a CFX96 Real-Time PCR System (Bio-Rad, USA) with SYBR Green PCR Master Mix (TransGen Biotech, China). *Actin* served as the internal control. The RT-qPCR primers used are detailed in Table S4.

### RNA-seq and data analysis

The WT and amiR-*SlRBP1* fruit at 25 DPA were utilized as samples for RNA sequencing, with three biological replicates per sample. The RNA-seq libraries were prepared and sequenced at Majorbio (Shanghai, China). For data analysis, clean reads were aligned to the Tomato reference genome (version SL4.0) using Hisat2 [49], and annotated according to ITAG4.0. DEGs were identified using DESeq2, applying the thresholds of |fold change (FC)| > 2 and an adjusted *P*-value <0.05 [50]. GO annotation and KEGG analysis of genes in clusters was performed using TBtools [51]. The dot plot and cluster volcano plot were generated using https://www.bioinformatics.com.cn [52].

### Protein extraction and western blot

Total proteins were extracted from WT and OE-*SlRBP1* 25DPA tomato fruits following a previously established protocol [24]. The antibodies against Myc tag (Sigma-Aldrich, MO, USA), Actin (Abmart, China), SleIF4A (Agrisera, Vannas, Sweden) and His (Abmart, Shanghai, China) were used for Western blot. All the above antibodies were diluted at 1:5000. Imaging was performed with Tanon-5200 (Tanon Science & Technology, China) after using ECL luminescent solution (Absin, Shanghai).

### Pull-down assays

The pET46–6× His-SlRBP1 vector was generate for His-SlRBP1 purification. The constructed vectors were transformed into *Escherichia coli* strain Rosetta (DE3) to facilitate the recombinant His-SlRBP1 fusion proteins *in vitro*. The method of His-SlRBP1 purification is described as previously [53]. Five microgram of purified His-SlRBP1was added to 700 μl of binding buffer. After incubating at 4°C for 5 minutes, Dynabeads™ His-tag Isolation and Pull-down beads (Thermo Fisher Scientific, MA, USA) were added to incubated for 30 minutes. The total RNA of 25 DPA fruit was folded at 95°C for 2 minutes and then added to the pull-down buffer. The folded RNA and magnetic bead-His-RBP1 complex was incubated at 4°C for 30 minutes. The His-SlRBP-RNA complexes were eluted at 4°C for 5 minutes. The enriched RNAs were recovered after digestion by protease K (Thermo Fisher Scientific, USA). After the extracted RNA was reverse-transcribed (Vazyme, China), the SlRBP1 target genes was verified by PCR 2× Taq Master Mix (Vazyme, China).

### nRIP-seq and nRIP-qPCR

Native RNA immunoprecipitation was carried out following a previously established protocol, with certain adjustments made to the procedure [28]. Fruit pericarp of 25 DPA OE-*SlRBP1* fruits were extracted by lysis buffer. The supernatant incubated with Myc Magnetic Beads at 4°C for 2 hours (Thermo Fisher Scientific, USA).The eluted SlRBP1-RNA complexes were treated with proteinase K (Thermo Fisher Scientific, USA) and DNase I (TransGen Biotech, China) at 50°C for 10 minutes before RNA extraction.

For nRIP-seq, RNA library preparation and sequencing were performed by Novogene (Beijing, China). High-quality reads were subsequently mapped to the tomato reference genome (SL4.0) using BWA (v0.7.17) [54]. The binding regions targeted by SlRBP1 were identified through peak calling by MACS2 software (version 2.2.8) with ‘-f BAMPE--nomodel-keep-dup all -B’ [55]. A stringent false discovery rate cutoff of *q* < 0.05 was applied to identify high-confidence binding regions for both WT and OE-SlRBP1. The overlapping peaks were analyzed using the intersectBed function from BEDtools [56]. Annotate the calling peaks by R (version 3.6.0) [57]. For nRIP-qPCR, input and immunoprecipitate RNAs were reverse transcribed (TransGen Biotech, China) and then analyzed by RT-qPCR.

### Statistical analysis

Data significance was assessed via SPSS v20.0, employing Student’s t-test for pairwise comparisons. For three or more data sets, pairwise comparison of data sets was performed using one-way ANOVA. A significant level was set at a *P* < 0.05.

### Accession numbers

Sequence data associated with this study are accessible through the Sol Genomics Network database (https://solgenomics.sgn.cornell.edu/). Table S5 contains the accession numbers of this article mentioned.

## Supplementary Material

Web_Material_uhaf089

## Data Availability

The raw sequence data of RNA-seq and nRIP-seq reported in this paper have been deposited in the Genome Sequence Archive [58] in National Genomics Data Center [59], China National Center for Bioinformation / Beijing Institute of Genomics, Chinese Academy of Sciences (GSA: CRA019577 and CRA019587) that are publicly accessible at https://ngdc.cncb.ac.cn/gsa. All data generated or analyzed during this study are included in the manuscript and supporting files (Figs S1–S7; Tables S1–S5).
