# Supplementary material for: A SlRBP1-SlFBA7/SlGPIMT module regulates fruit size in tomato
Source: Hortic Res. 2025 Mar 18;12(7):uhaf089. doi: 10.1093/hr/uhaf089 (PMC12064952; doi:10.1093/hr/uhaf089)
Supplement: Web_Material_uhaf089 [file web_material_uhaf089.zip › Supplemental data-clean.doc]

**Article title**: A SlRBP1-*SlFBA7/SlGPIMT* module regulates fruit size in tomato

The following Supporting Information is available for this article:

**Fig. S1** The expression pattern and identification of *SlRBP1* in T0 generation lines.

**Fig. S2** Silencing or over-expressing *SlRBP1* using the PPC2 promoter had no effect on plant vegetable growth.

**Fig. S3** Silencing *SlRBP1* does not affect fruit ripening, sugar and acid content.

**Fig. S4** Determination of the DNA ploidy level in WT, OE-*SlRBP1* and amiR-*SlRBP1* 25 DPA fruits.

**Fig. S5** Determination of the hormone level in WT, OE-*SlRBP1* and amiR-*SlRBP1* 25 DPA fruits.

**Fig. S6** Heat map showing the expression pattern of SlRBP1 targets.

**Fig. S7** Expression of *SlRBP1* in T1 overexpressing *SlRBP1* transgenic plants.

**Table S1** List of DEGs in amiR-*SlRBP1* compared with WT.

**Table S2** List of SlRBP1 29 target RNAs in fruit.

**Table S3** Alternative splicing analysis of WT and amiR-*SlRBP1* 25 DPA fruit transcriptomic data.

**Table S4** List of primers used in this study.

**Table S5** List of gene accession used in this study.

**Fig. S1** The expression pattern and identification of *SlRBP1* in T0 generation lines. (a) RT-qPCR assay shows expression levels of *SlRBP1* in different tissues and fruit development stages of tomato. Relative expression of *SlRBP1* in stem was set to 1. (b) The relative expression levels of *SlPPC2* in different tissues and fruit development stages of tomato. Relative expression of *SlPPC2* in stem was set to 1. (c) Relative expression of *SlRBP1* in T0 generation over-expressed or artificial miRNA silenced transgenic plants at MG fruit. Relative expression of *SlRBP1* in WT was set to 1. *Actin* was used as a reference gene. Error bars indicate ±SD over three biological replicates. Different letters indicate statistically significant differences as determined by One-way ANOVA (P<0.05). (d) Western Blot assay shows protein levels of SlRBP1 in WT and independent T0 OE-*SlRBP1* lines. C-Myc antibody was used to detect the fusion proteins.

**Fig. S2** Silencing or over-expressing *SlRBP1* using the PPC2 promoter had no effect on plant vegetable growth. (a-d) Representative pictures of 2-week-old plants (a), 5-week-old plants (b), flower size (c) and inflorescence (d). The Scale bars are shown in different graphs. (e) Final plant height of WT, OE-*SlRBP1* and amiR-*SlRBP1*. (f-h) The expression of *SlRBP1* in stem (f), leaf (g) and flower (h) of WT, OE-*SlRBP1* and amiR-*SlRBP1* lines. *Actin* was used as a reference gene. Relative expression of *SlRBP1* in WT was set to 1. Error bars indicate ±SD over three biological replicates. Statistical analysis was determined by One-way ANOVA (ns, not significant).

**Fig. S3** Silencing *SlRBP1* does not affect fruit ripening, sugar and acid content. (a) Observation of the maturation process of WT, OE-*SlRBP1* and amiR-*SlRBP1* fruits. (b) Statistics of breaking time of WT, OE-*SlRBP1* and amiR-*SlRBP1* fruits. n=50. (c) Soluble solids content in WT, OE-*SlRBP1* and amiR-*SlRBP1* fruits at different stages. (d) Titratable acid content in WT, OE-*SlRBP1* and amiR-*SlRBP1* fruits at different stages. MG, mature green; Br, Breaker; B+3, three days after breaker; B+6, six days after breaker; B+9, nine days after breaker. Error bars in (c) and (d) indicate ±SD over nine biological replicates. Statistical analysis was determined by One-way ANOVA (*P < 0.05; ns, not significant).

**Fig. S4** Determination of the DNA ploidy level in WT, OE-*SlRBP1* and amiR-*SlRBP1* 25 DPA fruits. (a-c) Ploidy-level distribution of wild type (a), OE-*SlRBP1* (b) and amiR-*SlRBP1* (c) lines. Approximately 10,000 nuclei were counted for each sample. Data represents mean ± SD of three independent replicates. (d) The percentage of different ploidy cells of each line fruit.

**Fig. S5** Determination of the hormone level in WT, OE-*SlRBP1* and amiR-*SlRBP1* 25 DPA fruits. The content of IAA (a), GA1 (b), GA3 (c), GA4 (d) and TZ (e) in WT, OE-*SlRBP1* and amiR-*SlRBP1* 25 DPA fruits. Data represents mean ± SD of three independent replicates. Statistical analysis was determined by One-way ANOVA (*P < 0.05; ns, not significant).

**Fig. S6** Heat map showing the expression pattern of SlRBP1 targets that have been validated for binding both *in vitro* and *in vivo* in tomato.

**Fig. S7** Expression of *SlRBP1* in T1 overexpressing *SlRBP1* transgenic plants. (a) RT-qPCR assay shows expression levels of *SlRBP1* in WT and different OE lines. *Actin* was used as a reference gene. Relative expression of *SlRBP1* in WT1 was set to 1. Error bars indicate ±SD over three biological replicates. Statistical analysis was determined by One-way ANOVA (*P < 0.05; ns, not significant). (b) Western Blot assay shows protein expression levels of FMF-SlRBP1 in WT and different T1 OE-*SlRBP1* lines. C-Myc antibody was used to detect the fusion proteins. Actin as internal reference protein.

**Table S1** List of DEGs in amiR-*SlRBP1* compared with WT. this table was submitted separately.

**Table S2** List of SlRBP1 29 target RNAs in fruit.

| **Supplemental Dataset S2. List of of SlRBP1 29 target RNAs in fruit.** | | | | | | |
| --- | --- | --- | --- | --- | --- | --- |
| Gene ID | Description | Enrichment Replicate 1 | Enrichment Replicate 2 | RNAseq_baseMean | log_2_FoldChange | padj |
| Solyc00g021640 | Ribosomal protein S4 | 8.94 | 7.15 | 33.45 | -0.435 | 0.53 |
| Solyc01g102310 | Unknown Protein | 43.78 | 42.37 | 8234.01 | -0.007 | 0.98 |
| Solyc01g104720 | Unknown Protein | 8.19 | 16.86 | 62292.59 | -0.118 | 0.51 |
| Solyc01g108500 | Polyadenylate-binding protein | 5.67 | 6.13 | 25447.65 | -0.351 | 0.04 |
| Solyc02g062240 | Small nuclear ribonucleoprotein SmD3 | 160.33 | 133.22 | 652.26 | 0.162 | 0.53 |
| Solyc02g077880 | Auxin-repressed protein | 6.19 | 7.15 | 70774.63 | -0.120 | 0.60 |
| Solyc02g082000 | 30S ribosomal protein S19 | 24.88 | 62.27 | 6456.66 | -0.310 | 0.07 |
| Solyc02g083260 | GPI mannosyltransferase 1 | 29.29 | 11.23 | 707.00 | 0.194 | 0.46 |
| Solyc02g084430 | COL domain class transcription factor | 5.35 |  | 104.62 | 0.147 | 0.83 |
| Solyc03g043600 | E3 ubiquitin-protein ligase SINA-like 10 | 25.11 | 56.66 | 1865.16 | -0.170 | 0.33 |
| Solyc03g078400 | MYB transcription factor-like | 5.35 | 5.61 | 2569.99 | 0.349 | 0.06 |
| Solyc03g096880 | Unknown Protein | 22.99 | 13.27 | 748.37 | -0.317 | 0.22 |
| Solyc03g116220 | Unknown Protein | 6.19 | 7.15 | 4431.54 | -0.992 | 0.07 |
| Solyc03g116230 | Agglutinin isolectin I | 6.54 | 6.13 | 3488.61 | -1.255 | 0.27 |
| Solyc05g054690 | Unknown Protein | 16.69 | 17.35 | 23.23 | 1.234 | 0.12 |
| Solyc06g076570 | class I heat shock protein | 4.13 | 6.13 | 4029.90 | -1.464 | 0.01 |
| Solyc07g064830 | 1 4-alpha-glucan branching enzyme | 76.29 | 95.45 | 684.79 | -1.046 | 0.00 |
| Solyc09g009260 | Fructose-bisphosphate aldolase | 4.47 | 7.15 | 8464.12 | 0.491 | 0.27 |
| Solyc09g010870 | Exoribonuclease R/ribonuclease II | 13.86 | 11.23 | 2125.49 | -0.355 | 0.04 |
| Solyc09g090710 | Unknown Protein | 20.30 | 11.74 | 499.41 | 0.743 | 0.00 |
| Solyc09g092380 | Adenosylhomocysteinase | 18.58 | 10.21 | 26502.26 | 0.065 | 0.84 |
| Solyc10g075090 | Non-specific lipid-transfer protein | 9.76 | 9.98 | 14756.84 | 2.012 | 0.00 |
| Solyc10g080780 | BZIP transcription factor | 46.93 | 17.35 | 969.09 | -0.035 | 0.89 |
| Solyc10g081170 | Calmodulin-2 | 4.13 | 8.68 | 30706.11 | -0.288 | 0.73 |
| Solyc10g081570 | KED | 8.26 | 21.95 | 16377.37 | -1.042 | 0.44 |
| Solyc11g007080 | YIPF5 | 75.60 | 59.72 | 583.92 | -0.204 | 0.34 |
| Solyc11g066830 | Splicing factor U2af subunit | 5.67 | 17.87 | 5090.52 | 0.055 | 0.78 |
| Solyc12g014270 | Peptide-N4-(N-acetyl-beta-glucosaminyl)asparagine amidase A | 179.55 | 191.41 | 81.18 | 0.750 | 0.36 |

**Table S3** Alternative splicing analysis of WT and amiR-*SlRBP1* 25 DPA fruit transcriptomic data. this table was submitted separately

**Table S4** List of DEGs in amiR-*SlRBP1* compared with WT. this table was submitted separately

| **Supplemental Table S4. Primers used in this study.** | | | |
| --- | --- | --- | --- |
| Gene | Primer | Sequence (5’-3’) | Experiment |
| *SlPPC2 promoter* | For | gcttgcatgcctgcagCATTCTACTTTGAAGTTGTTTAATGAGG | Recombinant OE-*SlRBP1* vector construction |
|  | Rev | tgtagtccatggatccACTAGAAAATCAAAATCCTGCAAAAAAT |  |
|  | For | gccaagcttgcatgcctgcagCATTCTACTTTGAAGTTGTTTAATGAGG | Recombinant amiR-*SlRBP1* vector construction |
|  | Rev | attcttctctactttggatccACTAGAAAATCAAAATCCTGCAAAAA |  |
| *SlFBA7* | For | ggactctagaggatccATGTCTTGCTACAAGGGAAAGTATG | Recombinant OE-*SlFBA7* vector construction |
|  | Rev | ctttgaatcgggatccGTACTTGTAGTCCTTAACATGAAGGCTCT |  |
|  | For | GAGAACACGGGGGACTCTAGAGGATCCAAAGTAGAGAAGAATCTG | Recombinant amiR-*SlFBA7* vector construction |
|  | Rev | CGATCGGGGAAATTCGAGCTCTGTAGTAAGAAGAGCCAA |  |
| *SlGPIMT* | For | ggactctagaggatccATGATGGTGACTGTAAATCTGAAGTCG | Recombinant OE-*SlGPIMT* vector construction |
|  | Rev | ctttgaatcgggatccTCTCTGTTTGACTTGTTTCTTTGAAGAGGC |  |
|  | For | GAGAACACGGGGGACTCTAGAGGATCCAAAGTAGAGAAGAATCTG | Recombinant amiR-*SlGPIMT* vector construction |
|  | Rev | CGATCGGGGAAATTCGAGCTCTGTAGTAAGAAGAGCCAA |  |
| *SlRBP1* | For | ATGGTTTTGCCGTGGTTAC | RT-qPCR for Relative expression of genes |
|  | Rev | GTAAACAAGGATCCACAGGGT |  |
| *SlPPC2* | For | GATCGGCACACTGATGTCCT |  |
|  | Rev | CAAATAGAGGTCGCTTGCCG |  |
| *SlXTH7* | For | TCCAACTTCGTCGTTCCACA |  |
|  | Rev | CACCAAGTGCTTCGTGGTTG |  |
| *SlXTH3* | For | TGACCCTACTGCTGATTTCCAC |  |
|  | Rev | TTGGCGTTCCGTCCACATAA |  |
| *SlXTHB1* | For | CTTCTCGCCTTGTCCCTTGA |  |
|  | Rev | GCCCTGCTCCTTGAGAAGAC |  |
| *SlAET* | For | TGCAGCATGGCTAGAGAAAATG |  |
|  | Rev | AATCTCTGGTTGGTGTCGCA |  |
| *SlLAC22* | For | ACCATGCAATCCTGGCATCT |  |
|  | Rev | TCGCAGTCCAAGATTTGGGA |  |
| *SlEXPA5* | For | GTTGGAGGTTCAGGGGATGT |  |
|  | Rev | CCGTTAAGGTAAGCGTTATTCT |  |
| *SlMYC2* | For | AGCAGGAGCATCGGAAGAA |  |
|  | Rev | CCAAATCGGGCTGGAACTA |  |
| *SlPhiP1* | For | ATTCCACCAGCCCATCTACG |  |
|  | Rev | CCAAATGGGTTTGTTGCGGT |  |
| *SlPOW1* | For | AGCTCCTTACACACGCCAAA |  |
|  | Rev | GCAGCTCCACCTAGCTTTCA |  |
| *SlActin* | For | TGTTGCTATCCAGGCTGTGCTTTCT |  |
|  | Rev | TCATGTCACGTACAATTTCCCGTTC |  |
| *SlFBA7* | For | GAGTACACCGTCCGTGCTTT |  |
|  | Rev | GTTGACGGTGGCTTCCTCTT |  |
| *SlGPIMT* | For | ACCAACATGAAGCTCGGAGG |  |
|  | Rev | CCAAGAACAGCAAACCTGCC |  |
| *SlActin* | For | CAGCAGATGTGGATCTCAAA | RIP-qPCR and protein pull-down assay |
|  | Rev | CTGTGGACAATGGAAGGAC |  |
| *Sltublin* | For | CCTGACAGCTTCTGCCATGT |  |
|  | Rev | CATCTTCAGCCCAGTTGGTG |  |
| *SlRIP1b* | For | CTAAAAGAGCTGCCTAAGGTTCG |  |
|  | Rev | AGGGTTCTGACTGGGTTGAT |  |
| *SlUn01* | For | TTCAGAGCCAGCTCCCATTG |  |
|  | Rev | ATTTCCGGTAACAGCCTCGG |  |
| *SlSmD3* | For | CTAAAGCTCAGGCAGCAGGT |  |
|  | Rev | AGGGAACAGAAGCGTATGCC |  |
| *SlFBA7* | For | GAGTACACCGTCCGTGCTTT |  |
|  | Rev | GTTGACGGTGGCTTCCTCTT |  |
| *SlGPIMT* | For | ACCAACATGAAGCTCGGAGG |  |
|  | Rev | CCAAGAACAGCAAACCTGCC |  |
| *SlBZIP* | For | CTCTACTGCGTTGTGGAGGG |  |
|  | Rev | GCCCACTTTCTCCCTGTTGA |  |
| *SlKED* | For | CAATGCCAAACTGGAAGCTCT |  |
|  | Rev | AGTCGTGTGCCTTCGATTCA |  |
| *SlYIPF5* | For | GGCAGAAACGGGAATTTGGAC |  |
|  | Rev | CACCAATCCACCAGGCAAAA |  |
| *SlMYB-L* | For | GCAATGGAGGCAAGTGAACC |  |
|  | Rev | ggaCCGGACAAGCTGGTAAA |  |
| *SlSINA-L-10* | For | GCTCATCTGGACTCATCGGG |  |
|  | Rev | CTAGTCAGCGGAGGCGAAAA |  |
| *SlARP* | For | CCTTCTCACCCTAACGCTCC |  |
|  | Rev | CCACCCAAAAACTCTCAGCAC |  |
| *SlLTP2* | For | ATACAGGAAAAGCCGCTGGG |  |
|  | Rev | GGACCCGTACGTTGAGCAAT |  |
| *SlGBE* | For | GTGGCCTTTTTGAGGTGAGT |  |
|  | Rev | CCGGATGCTGAAGGCAAAAG |  |
| *Slexp1* | For | GGAGTTAACACAGCAGCACT |  |
|  | Rev | TCCAGGAAGACACCATTTCCA |  |

**Table S5 List of DEGs in amiR-SlRBP1 compared with WT.**

| **Supplemental Table S5. The gene ID used in this study.** | |
| --- | --- |
| Gene_Name | Gene_ID |
| *SlRBP1* | Solyc01g109660 |
| *SlPPC2* | [Solyc07g062530](https://solgenomics.sgn.cornell.edu/tools/blast/match/show?blast_db_id=224;id=Solyc07g062530.2;hilite_coords=1-3357) |
| *SlXTH7* | Solyc07g056000 |
| *SlXTH3* | Solyc03g093130 |
| *SlXTHB1* | Solyc12g017240 |
| *SlAET* | Solyc01g057770 |
| *SlLAC22* | Solyc02g090360 |
| *SlEXPA5* | Solyc02g088100 |
| *SlMYC2* | Solyc08g076930 |
| *SlPhiP1* | Solyc04g074450 |
| *SlPOW1* | Solyc07g040960 |
| *SlActin* | Solyc03g078400 |
| *Sltublin* | Solyc04g081490 |
| *SlRIP1b* | Solyc12g014230 |
| *SlUn01* | Solyc01g102310 |
| *SlSmD3* | Solyc02g062240 |
| *SlFBA7* | Solyc09g009260 |
| *SlGPIMT* | Solyc02g083260 |
| *SlBZIP* | Solyc10g080780 |
| *SlKED* | Solyc10g081570 |
| *SlYIPF5* | Solyc11g007080 |
| *SlMYB-L* | Solyc03g121990 |
| *SlSINA-L-10* | Solyc03g043600 |
| *SlARP* | Solyc02g077880 |
| *SlLTP2* | Solyc10g075090 |
| *SlGBE* | Solyc07g064830 |
| *Slexp1* | Solyc06g051800 |
